# Prevalence and Risk Factors of Gestational Diabetes Mellitus among Women Attending Antenatal Care in Hadiya Zone Public Hospitals, Southern Nation Nationality People Region

**DOI:** 10.1155/2021/5564668

**Published:** 2021-04-05

**Authors:** Yilma Markos Larebo, Niggussie Abebe Ermolo

**Affiliations:** ^1^Department of Epidemiology, College of Medicine and Health Sciences, Wachemo University Hossana, Ethiopia; ^2^Department of Health Service Management, College of Medicine and Health Sciences, Wachemo University, Hossana, Ethiopia

## Abstract

**Introduction:**

In low- and middle-income countries, gestational diabetes mellitus is increasing globally; it is also a double burden of illness for both mothers and children. While gestational diabetes mellitus is recognized in Ethiopia, according to recent diagnostic criteria, information regarding it remains scarce.

**Objective:**

To assess the prevalence of gestational diabetes mellitus and associated factors among women attending antenatal care in Hadiya Zone public Hospitals, Southern Ethiopia.

**Methods:**

An institution-based cross-sectional research on a total of 470 pregnant mothers was conducted in the Hadiya Region from August 2019 to December 2020. Finally, via the systematic random sampling process, the study subjects were chosen. A two-hour oral glucose tolerance test of 75 g was used to conduct the universal one-step screening and diagnostic technique. Bivariate and multivariate analyses were used to identify factors associated with gestational diabetes mellitus.

**Results:**

Gestational diabetes mellitus prevalence was 26.2% (95% CI, 21.8, 30.5). Urban residents (AOR: 2.181; 95% CI: 1.274, 3.733), primary education (AOR:2.286; 95% CI: 1.396, 3.745), without previous history of abortion (AOR: 0.097; 95% CI: 0.048, 0.196), with history of late gestational age in weeks (29-32) (AOR: 0.393; 95% CI: 0.213, 0.723), with no history of coffee drinking (AOR: 2.704; 95% CI: 1.044, 7.006), and adequate dietary diversity (AOR: 2.740; 95% CI: 1.585, 4.739) were significantly associated with gestational diabetes mellitus.

**Conclusion:**

In Hadiya Zone public Hospitals, the prevalence of gestational diabetes mellitus among women attending antenatal treatment was higher compared to other studies conducted. The urban residents, primary schooling, no prior history of abortion, late gestational age, no history of coffee drinking, and sufficient dietary diversity were significantly linked with gestational diabetes mellitus. To enhance maternal and child health, reinforcing screening, treatment, and prevention strategies for gestational diabetes mellitus is essential.

## 1. Introduction

Gestational diabetes mellitus (GDM) is generally characterized as glucose intolerance that changes the degree of severity that begins or is first detected during pregnancy, usually after the 24th week of gestation [1–4]. It is also known as intolerance to carbohydrates resulting in variable severity hyperglycemia with onset or first recognition during pregnancy [5].

Diabetes diagnosed in the second or third trimester of pregnancy has been ruled out because of overt diabetes early in pregnancy and is not preexisting type 1 or type 2 diabetes [6, 7]. Gestational diabetes mellitus occurs either when the pancreas does not produce enough insulin or when the insulin it produces cannot be used efficiently by the body. Insulin is a blood sugar-regulating hormone [8]. Symptoms include blurred vision; fatigue; regular infections like bladder, vagina, and skin; increased vision like thirst, appetite, and urination; nausea and vomiting; and loss of weight [9].

The global effect of gestational diabetes is growing and both mothers and infants are doubly burdened by the disease. The prevalence in the general population compares with the pregnancy rate [10, 11]. It affects up to 1 out of 7/10 pregnancies worldwide and, in combination with other noncommunicable diseases (NCDs), accounts for 70% of all deaths worldwide [1, 7, 12].

The global prevalence of all births can range from 2.4 to 21% [12, 13]. The prevalence was approximately 16.9 percent among women in the reproductive age group [1, 2]. This varies greatly depending on the studied population and the diagnostic test used [12].

The burden is rising in low- and middle-income countries, with some 90% of cases occurring in developed countries. The estimated total prevalence in Africa was 5% [1, 7, 12]. It also makes about 4% of all pregnancies difficult and women with it have an estimated 7-fold chance of developing type 2 diabetes in the future, as well as their children and subsequent generations [10, 14].

While diabetes mellitus is recognized as one of the major chronic diseases in Ethiopia, the prevalence ranges from 4 to 13% for NCDs [1]. Increased risk of preeclampsia in mothers and increased risk of macrosomia, hypoglycemia, jaundice, respiratory failure, polycythemia, and hypocalcemia in newborn babies. There is postpartum progression if no treatment is needed [10, 11, 15].

Because of postpartum development, women with GDM are advised to be screened for type 2 diabetes 4-12 weeks postpartum and referred for follow-up if diabetes is identified [16]. Therefore, early diagnosis of GDM is important for prevention [6].

In our country, the prevalence of GDM among pregnant mothers and factors associated with it have not been well researched. There is no research on gestational diabetes mellitus and associated risk factors, especially in the field of study up to the investigator's knowledge. Because of all these causes, the consciousness of the community about the conditions is low.

It will be necessary to recognize the prevalence of the problem and common risk factors to mitigate the problem on a timely basis and to promote health policy and enhancement of the program. Therefore, the purpose of the study was to evaluate the prevalence and associated risk factors of gestational diabetes mellitus among pregnant mothers in the Hadiya region of southern Ethiopia; besides, the findings will be used as a guideline for those interested in researching the same subjects.

## 2. Methods and Materials

### 2.1. Study Setting

The study was conducted in Hadiya Zone public hospitals among a cohort of pregnant mothers recruited from the general population attending antenatal care in public hospitals of the Zone. The Zone was found in Southern Nation Nationality People Regional State (SNNPR). The Zone is located in South West of Ethiopia, 230 km far away from Addis Ababa, the capital city of Ethiopia, and 194 km from the regional capital city, Hawassa.

Administratively, the Hadiya Zone was organized by 4 administrative towns, 13 districts, 305 rural Kebeles, and 30 urban Kebeles, and estimated population size of 1,727,920 with male 856,357 (49.56%) and female 871,563 (50.44%). Estimated number of reproductive age mothers in the Zone, which was about 402,605 (23.3%), which comprises an estimated 23,155 (3.46%), pregnant mothers, from age 18 to 49 years on study area based on 2007 census conversion factor projection and have a population density of 92 inhabitants per km^2^ [17, 18].

In the Zone, there were a total of 376 health institutions from this; there is 1 general hospital, 3 primary hospitals, 3 primary hospitals (under construction), 61 health center, 311 health posts, and 81 private clinics (1 higher, 16 medium, and 64 lower) and 39 private pharmacies (2 pharmacies, 17 drug stores, and 20 rural drug vendors), which would deliver routine health services to the community. Health coverage was not yet satisfied, and all health facilities were not currently providing blood glucose level tests for GDM patients [17–19].

### 2.2. Study Design and Period

From August 2019 to December 2020, an institution-based cross-sectional study design was carried out in public hospitals of the Hadiya Region among a cohort of pregnant mothers recruited from the general population attending antenatal care in public hospitals of the Zone.

### 2.3. Source Population

The source population was all pregnant mothers aged 18-49 years living in the Zone.

### 2.4. Study Population

The research population of all selected pregnant mothers with 24-32 weeks of gestational age living in the Zone.

#### 2.4.1. Sample Size Determination

The sample size was calculated using single population proportion formula, considering the following assumptions and taking the prevalence of gestational diabetes mellitus 12.8% which was a study conducted in Northwest Ethiopia [20].

(1) $$\begin{array}{c} n=\frac{{\left(Z\alpha /2\right)}^{2}p\;\left(1-p\right)}{d^{2}}, \end{array}$$

where *n* is the desired sample size, *P* is the prevalence of gestational diabetes mellitus (12.8%) (which was taken from a study conducted at Gondar town public health facilities, Northwest Ethiopia, 2019), *Z*1 − *α*/2 is the critical value at 95% confidence level (1.96), *d* is the margin of error (5%), *n* = ((1.96)^2.^0.128(1 − 0.128)(/(0.05)^2^ = 172. For possible none response during the study, the final sample size was increased by10% to *n* = 172 + 10% of 172 which is 17.2, by adding; then, the total sample size was 189.

### 2.5. The Sample Size for Second Objectives

Since the sample size calculated for the second objectives was larger than the sample size calculated for the first objectives, so the sample size of 470 was a sample size of the study, where *P* is the percent of outcome in unexposed groups ratio, unexposed to exposed OR (odds ratio), odds of exposed to unexposed and power, and the probability of rejecting the null hypothesis when it is false (see Table 1).

### 2.6. Sampling Procedure

From the total hospitals offering treatment and care for pregnant mothers with gestational diabetes in the Zone, mothers with a gestational age of 24-32 weeks who attended antenatal care in selected 1 general and 3 primary hospitals were selected deliberately [18].

**Figure 1:**
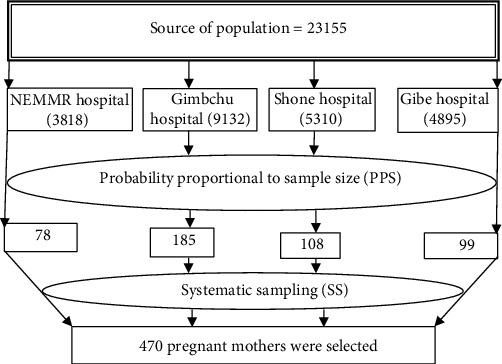
Schematic presentation of sampling procedure gestational diabetes mellitus among the pregnant mothers in Hadiya Zone public hospitals, Southern Ethiopia: August 2019-December 2020 (*n* = 470).

Based on their source population from each hospital, the total sample size was allocated proportionately to the four public hospitals, and pregnant women who met the inclusion criteria were selected before data collection by performing a census in the selected hospitals; then, eligible respondents were registered by reviewing antenatal care records, and a code number was issued to eligible respondents. In the chosen hospitals, 23,155 pregnant mothers were between the ages of 18 and 49 years [18]. The research participants were then randomly selected for antenatal care (ANC) follow-up from each hospital and pregnant mothers, who were eligible for the study until the total required sample sizes were obtained (see Figure 1).

### 2.7. Study Variables

#### 2.7.1. Dependent Variables

The dependent variable is gestational diabetes mellitus (1: yes, 0: no).

#### 2.7.2. Independent Variables

Independent variables are as follows:

1. Sociodemographic-related characteristics of respondents like age, sex, marital status, occupation mother, education mother, religion, income, and residency
2. Obstetric- and clinical-related factors like the birth weight of the previous child, family history of diabetes mellitus(DM), previous history of GDM, family history of type II DM, middle upper arm circumference (MUAC), blood pressure (BP), blood glucose level, hemoglobin, previous cesarean section, history of having a macrocosmic baby, and gestational age in weeks
3. Behavioral- and lifestyle-related characteristics of respondents like antenatal depression, alcohol drink, coffee drink, physical activity, and dietary diversity

### 2.8. Data Collection Procedures

A questioner was used to collect quantitative data using a standardized interviewer-administered questionnaire to test gestational diabetes mellitus. The questionnaire was prepared in English and then translated back to English to verify the accuracy in the local language Hadiyisa.

### 2.9. Instruments and Measurements

To be understood by all respondents and back-translated to English as interviewers proceeding consecutively from one pregnant mother to another, the data was translated into the Hadiyisa language. Eighteen (4 laboratory technologists (supervisors), 14 data collectors (6 laboratory technologists, 6 Bachelor of Science (BSc) nurses, and 2 clinical nurses) were recruited and trained by Princ for two days. For 12 working days, they collected data (from August 2019-December 2020). In a typical process, they introduced themselves and explained the intent of the study using clear statements. Each interviewee obtained the consent of the study participants.

For the data collection, predefined and prestructured proforma was used. The capillary blood sample was taken two hours later by pricking with a lancet, and the amount of blood glucose was measured and recorded on the spot by a glucometer. The dating ultrasounds were performed by trained and experienced clinical data collectors to confirm gestational age if necessary.

The gestational age and predicted date of birth were based on the theory of Naegele and the fundal height palpation. If the last date of the menstrual cycle was unclear or there was a difference between the two parameters, then an obstetric ultrasound was requested and the ultrasound result was dependent on the gestational era.

### 2.10. Inclusion and Exclusion Criteria

All pregnant mothers were enrolled between the ages of 18 and 49 years between the gestational age of 24-32 weeks who were attending antenatal care services in selected hospitals during the survey period. But pregnant women documented who had cases of diabetes, multiple gestation or incomplete plasma glucose levels, unexplained prepregnancy body mass index, in labor, or patients with chronic diseases such as tuberculosis, malignancy, renal failure, congestive heart failure, advanced liver failure, and serious illness during data collection were removed.

#### 2.10.1. Operational Definitions

1. Gestational diabetes mellitus (GDM): the diagnosis of gestational diabetes mellitus was made, when 75 gm of glucose load and measurement of blood sugar level after 2 hours, ≥140-199 mg/dl [8, 12, 21]
2. Gestational age (GA) is the age of the fetus counting from the time of fertilization [22]
3. Parity is the number of live-born children a woman has delivered

- Primipara: those who gave birth only once
- Multipara: those who gave birth above one time
- Grand-multi: those who gave birth above five times

(4) Dietary diversity: it was tested by the Food and Nutrition Technical Assistance (FANTA) 2016 edition of the minimum dietary diversity measurement tool of a woman using a 24 h food recall process. A list of ten food groups was issued (starchy staples, nuts and seeds, pulses, dairy, meat, eggs, poultry and fish, dark green leafy vegetables, other vitamin-A rich fruits and vegetables, other vegetables, and other fruits). The minimum dietary diversity score (MDDS) was dichotomized based on whether or not women had eaten the preceding day or night list of specified food groups. The MDDS of five and more was rated as sufficient diversity of diets [23]
(5) Drink coffee: the mother was classified as exposed to coffee if pregnant mothers drank coffee “daily” or “sometimes in a week”
(6) Drink alcohol: the mother was labeled as exposed to alcohol if pregnant mothers consume alcohol “daily” or “sometimes in a week”.
(7) Mid-upper arm circumference: on the left arm, it was measured using a nonstretchable measuring tape. A ≥28 cm pregnant woman with MUAC was found to be overweight and/or obese [20]
(8) Hemoglobin: a pregnant woman with a concentration of hemoglobin below 11 g/dl was considered to have anemia [20]
(9) Blood pressure: the pregnant woman was asked to rest in sitting positions for at least 5 min if they were exercised. The pressure on the right arm was then measured using regular cuffs fitted with a standard sphygmomanometer for adults, positioning the stethoscope bell gently over the brachial artery. The mean systolic blood pressure (SBP) and diastolic blood pressure (DBP) were reported in mmHg after two measurements were taken at 5-10 min intervals. If the systolic and diastolic blood pressures were higher than or equal to 140 mmHg and 90 mmHg, respectively, hypertension was assumed to be present [20]
(10) Antenatal depression: using the Edinburgh Postnatal Depression Scale (EPDS) screening instrument developed and validated in urban Ethiopia, symptoms were measured. The system was used to quantify the emotions a mother had encountered in the past week. The tool includes 10 basic questions with four choices for Likert scale answer (most of the time, often, not always, never), graded from 0 to 3 (more depressive symptoms suggested by a higher score), which is easy to use and can be scored by simple addition. Similar studies conducted in Ethiopia and abroad used an EPDS score of 13 and more to categorize the presence of antenatal depression [24]

### 2.11. Physical Activity

The International Physical Activity Questioner (IPAQ) will be used as part of their daily lives to test the physical activities that women (15-49) do. It will be built to determine particular types of activities, such as walking, moderate and intense activities of intensity undertaken at work, as part of house and yard work, to get place to place, and in spare time for leisure, exercise or sport (last 7 days preceding the interview). Data will be recorded as metabolic equivalents (MET-minutes per week) for women in high, moderate, and low levels of physical activity groups using the IPAQ scoring protocol [9].

### 2.12. Data Quality Assurance

To ensure the consistency of the data, the nature of the data collection tool was emphasized for its simplicity and uniform group rating scales, validity, and reliability were taken into account and data collectors were educated. To check the accuracy, the questionnaire was prepared in English and then translated into the local Hadiya language and back-translated into English. To check the accuracy, the questionnaire was pretested on 24 mothers in Worabe Hospitals outside the study area, and the interview was carried out in private. Throughout the collection of data, interviewers were tracked at each location, daily meetings were held between the data collectors, the supervisor, and the principal investigator in which concerns resulting from interviews performed, and errors discovered during editing were addressed and decisions were made. Two further additional visits were made if the first visit did not find a respondent. Until data entry, the collected data were inspected and tested for completeness; incomplete data was discarded. The prototype for the data entry format was developed and programmed.

### 2.13. Data Processing and Analysis

Data were tested, coded, and entered in EPI Data version 3.1 and exported for analysis to Statistical Package for Social Sciences (SPSS) version 26. The key investigator was responsible for data entry. The variable description was performed and presented in frequency, using tables, graphs, charts, and chi-square statistics (*χ*^2^). Adjusted odds ratios (AOR) and a 95 percent confidence interval using logistic regression were used to verify the existence and intensity of the correlation between independent and dependent variables. In the bivariate analysis, variables having *P* values less than 0.25 were entered into the multivariate analysis using backward elimination. The fitness of the model was tested using the 0.796 Hosmer and Lemeshow test. Based on their relationship of importance (i.e., *P* < 0.05), the final result was interpreted.

## 3. Results

### 3.1. Characteristics of Respondents

Out of the 470 pregnant mothers invited to participate in the study, 50 mothers (22 did not return for oral glucose tolerance test (OGTT), 12 did not complete the tests, 9 were diagnosed with overt diabetes, 5 have a medical emergency, and 2 had an abortion before OGTT) were excluded and making the nonresponse rate of 10.6%.

Out of 420 women included in the study, almost half the 210 (50%) respondents were urban residents. The majority, 173(41.2%), were 18-25 years old. The mean age was 29.57 (7), the majority, 280(66.7%) were married and the majority of them having monthly income was less than 1500 Ethiopian Birr (ETB), 259 (61.7%). Most of the mothers, 190 (45.2%), were from Hadiya ethnic group and 274 (65.2%) had attended secondary and above education and attended secondary and above education was the leading educator of their partners, 350 (83.3%). The majority, 300 (71.4%), were housewives, and working as a government employee was the leading occupation of their partners, 239 (56.9%) (see Table 2).

Basic obstetric characteristics were assessed in this study. Screening of GDM was carried out at 24-32 gestational weeks. Of the 420 study participants, the majority, 180 (42.8%), had two or more deliveries, with a mean gestational age of 27.28 weeks. Nearly one-third of the respondents, 126(30%), were multigravida. Out of 420, 253 (60.2%) mothers who had no family history of DM, 320 (76.2%) with no previous history of GDM, 253 (60.2%) with no family history of type II DM, 300 (71.4%) had no previous history of stillbirth, 238(56.6%) had the previous history of abortion, 204 (48.6) with history of systolic/diastolic blood pressure, and cesarean section rate was 326 (77.6%).

Of the total 420 pregnant women, 53 (12.6%) had macrocosmic babies, 167 (39.7%) history of preterm labor, 73 (17.4%) mothers with a history of underweight childbirth, 302 (71.9%) history of gestational age between 24 and 28 weeks, 111 (26.4%) history of anemia, and 188 (44.8%) with history of overweight/obesity (see Table 3).

Out of 420 total participants, a low level of physical activity was reported by 380 (90.5%), 30 (7.1%) of the pregnant women had antenatal depression symptoms, 73 (17.4%), mothers with a history of alcohol intake, and majority of mothers in our study with a history of coffee intake and were 26 (6.2%) with history of chat chewing (see Table 4).

### 3.2. Prevalence of Gestational Diabetes Mellitus

**Figure 2:**
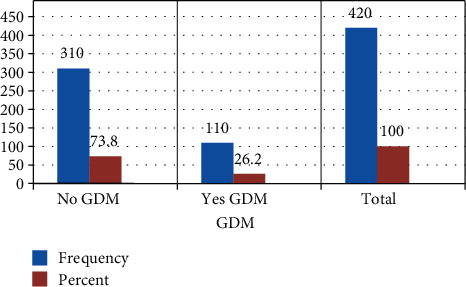
The prevalence of gestational diabetes mellitus of the study participants attending antenatal care at Public Hospitals in Hadiya Zone, Southern Ethiopia: August 2019-December 2020 (*n* = 420).

The overall prevalence of gestational diabetes mellitus was 26.2% of the respondents with 95% CI (21.8, 30.5) with a mean of 0.26 and standard deviation of ±0.44 (see Figure 2).

Multivariable analysis was used to control potential confounders. Accordingly, urban residence (AOR: 2.181; 95% CI: 1.274, 3.733), primary education (AOR: 2.286; 95% CI: 1.396, 3.745), mothers with no previous history of spontaneous abortion (AOR: 0.097; 95% CI: 0.048, 0.196), late gestational age from 29 to 32 weeks (AOR: 0.393; 95% CI: 0.213, 0.723), mothers with no history of coffee drink (AOR: 2.704; 95% CI; 1.044, 7.006), and adequate dietary diversity (AOR: 2.740; 95% CI: 1.585, 4.739) were found to be independently associated (see Table 5).

## 4. Discussion

The primary goal of this study was to determine the prevalence of GDM in the Hadiya Region of Southern Ethiopia, as well as to establish risk factors. 470 pregnant women between the ages of 24 and 32 weeks were screened for gestational diabetes mellitus using the World Health Organization's (WHO) 2013 guidelines.

The overall prevalence of gestational diabetes mellitus was 26.2%, 210 (50%) among urban and rural residents. This was found to be 2-fold higher than the previous point estimate of a study conducted in urban women in Tigray, Northern Ethiopia [25], and Gondar, Northwest of Ethiopia [20] and 6-fold higher southern Ethiopia [1]. It also four times high than the study that conducted a systematic review and meta-analysis among adults in Ghana which was 6.48% [26]. The prevalence of GDM varies across populations, ranging from 10.4 to 25% across the world [7].

The prevalence of gestational diabetes mellitus one and half times more than the study conducted in the prevalence of the study in Australia which was 17.8% [27]. Almost nearly similar to the study conducted by WHO 2013 Gestational Diabetes Mellitus Criteria Identify Obese Women with Marked Insulin Resistance in Early Pregnancy. Almost two times high than the study conducted in Israel which was 55.7% [28]. Over 90% of cases occur in low- and middle-income countries. This finding is somehow low than with studies conducted in Tanzania which was 29.9% [7], but higher than some other countries' studies like Tamil Nadu in Kancheepuram District which was 18.5% [29] and Rwanda which was 3.2% [12].

The variations between different countries in the prevalence of gestational diabetes mellitus can be attributed to differences in socioeconomic status, lifestyle, and screening and diagnostic methods. Differences in screening techniques and the use of different diagnostic criteria have made it difficult to compare the GDM situation across countries; given this fact, our finding shows that in the region the severity of the problem is increasing.

In this study, an urban residence was two times more likely to develop GDM than from rural residences. This finding is similar to the findings in Wolaita Zone, Southern Ethiopia [1]. It was also similar to the study conducted in Rwanda which was 4.28% [12], in contrast to this study, more common in those with rural residents than in urban residences is the study conducted in Gondar, Northwest Ethiopia [20].

In our sample, with mothers attending primary education, the proportion of gestational diabetes mellitus increased almost four times compared to secondary education and above; a similar finding was recorded in the study conducted in the wait region, southern Ethiopia [1]. This may be attributed to increased knowledge of diseases as the educational level of mothers rises.

In this study, GDM was less likely associated with pregnant mothers; the odds of developing GDM was 90.3% less likely among women with no previous history of abortion when compared with those who had a history of previous abortion. This result is inconsistent with other studies conducted in Wolaita Zone, Southern Ethiopia [1] and in Gondar, Northwest Ethiopia [20], and the study mentioned that previous history of spontaneous abortion was linked with an elevated possibility of acquiring GDM in Asia [30].

A pregnant mother with a history of large gestational age in weeks [22, 29, 31, 32], and 60.7% less likely was linked with the occurrence of GDM than small for gestational age. From other evidence, those mothers with increase gestational age have a higher risk of developing GDM [33].

Pregnant mothers with no history of coffee drink almost three times more likely to develop GDM than the coffee drinker was an independent predictor of gestational diabetes mellitus. Similar findings have been stated in studies conducted in Seattle, Tacoma, and Washington. Women who reported moderate prepregnancy caffeinated coffee intake had a significantly reduced risk of GDM (adjusted RR 0.50; 95% CI 0.29, 0.85) compared with nonconsumers [32]. Researchers at Harvard tracked over found that people who increased their coffee intake by over one cup per day had an 11% lower risk of developing GDM. However, people who reduced their coffee intake by one cup per day increased their risk of developing GDM by 17 percent. There was no difference in those drinking tea [29].

This may be explained by the consumption of decaffeinated coffee, also showing an immediate increase in blood sugar, or it could be that when you drink caffeine, it prevents the binding of adenosine receptors (AR) to your cells, allowing your cell activity to remain elevated, giving you more energy, and preventing you from falling asleep or increasing other chemicals that produce energy.

There was almost three times higher GDM than their counterparts for a pregnant mother with a history of sufficient dietary diversity. In comparison to the study conducted in Gondar, Northwest Ethiopia, and the intake of food from several dietary groups during pregnancy, this study is likely to trigger complications related to pregnancy [20]. This may be due to the influence of dietary variability that may increase the production of GDM during pregnancy.

The fact that the majority of women with GDM were listed as an adequate dietary diversity category may be the potential explanation for the correlation between adequate dietary diversity and GDM. Likewise, a significant proportion of women depended on the category of monotonous foods in which cereals were eaten most frequently. Refined carbohydrates and sugars were likely to have been excessive in their diets. Dietary diversity, on the other hand, ranged across a variety of variables linked to individuals' and households' demographic and socioeconomic status.

More research is needed to see whether improving dietary pattern adherence during pregnancy is linked to a higher risk of GDM. The rise in GDM would help clinical and public health initiatives to promote dietary diversity for women of reproductive age in potential births, according to our findings.

## 5. Conclusion

In Hadiya Zone public hospitals, the prevalence of gestational diabetes mellitus among women attending antenatal treatment was higher compared to other studies conducted. Urban residency, primary schooling, no prior history of abortion, late gestational age, no history of coffee drinking, and sufficient dietary diversity were significantly linked with gestational diabetes mellitus. To enhance maternal and child health, improving screening, treatment, and prevention strategies for gestational diabetes mellitus is necessary.

### 5.1. Recommendations for Future Research

The conduct of a stronger design review would have a better estimate to address the study's limitations.

### 5.2. Strength of the Study

The strength of the research was that it used a modern and universal screening method to detect GDM, and it was done at 24-32 weeks of gestation for all pregnant women. They underwent a two-hour 75 g OGTT, and consideration was given to modified standard reference cutoff values. Also, at late gestational age, pregnant women who had risk factors for GDM and whose OGTT results were negative during the daily test were checked again.

### 5.3. Limitations of the Study

The WHO recommends that the use of point-of-care tests may influence the outcome in settings where laboratories or proper storage and transport of blood samples are not guaranteed, as is the case in resource-limited countries such as Ethiopia. However, due to simplicity and reasonable reliability, we used plasma-calibrated handheld glucometers. Moreover, due to the nature of the research design, the causal inference was constrained and the temporal sequence between the variables and the outcome variable could not be disclosed, which could be a limitation of the study.

## Figures and Tables

**Table 1:** The sample size for the second objectives by using Epi Info version 7, on the prevalence of gestational diabetes mellitus among the pregnant mothers in Hadiya Zone public hospitals, Southern Ethiopia: August 2019-December 2020 (*n* = 470).

Variables	Assumption	Sample size
Previous cesarean section	OR = 7.5, *P* = 4.2%, ratio 1 : 1, power = 80%, CI = 95%	50 [1]
Family history of type II diabetes	OR = 6.2, P = 4.2%, ratio 1 : 1, power = 80%, CI = 95%	62 [1]
Previous history of spontaneous abortion	OR = 4.2, P = 4.2%, ratio 1 : 1, power = 80%, CI = 95%	114 [1]
Dietary diversity status	OR = 1.90, *P* = 12.8%, ratio 1 : 1, power = 80%, CI = 95%	470 [20]
MUAC	OR = 2.25, *P* = 12.8%, ratio 1 : 1, power = 80%, CI = 95%	438 [20]
Level of physical activity	OR = 3.36, P = 12.8%, ratio 1 : 1, power = 80%, CI = 95%	104 [20]
Family history of DM	OR = 4.03, *P* = 12.8%, ratio 1 : 1, power = 80%, CI = 95%	128 [20]
Antenatal depression	OR = 4.12, P = 12.8%, ratio 1 : 1, power = 80%, CI = 95%	124 [20]
Previous GDM	OR = 5.82, P = 12.8, ratio 1 : 1, power = 80, CI = 95%	76 [20]

**Table 2:** Selected sociodemographic characteristics of the study participants attending antenatal care at public hospitals in Hadiya Zone, Southern Ethiopia: August 2019-December 2020 (*n* = 420).

Variables	Categories	n (%)
Maternal age in years	≤25	173 (41.2)
	25 to 29	85 (20.3)
	30 to 34	63 (15)
	≥35	99 (23.6)
Spouse's education	Primary education	70 (16.7)
	Secondary education and above	350 (83.3)
Spouse's occupation	Government employee	239 (56.9)
	NGO employee	41 (9.8)
	Daily laborer	140 (33.3)
Religion	Orthodox	35 (8.3)
	Muslim	43 (10.2)
	Protestant	316 (75.2)
	Catholic	26 (6.2)
Monthly income in birr	<1500	259 (61.7)
	1500-2499	41 (9.8)
	2500-3999	39 (9.3)
	≥4000	81 (19.3)
Occupational status mothers	Housewife	300 (71.4)
	Government employee	94 (22.4)
	NGO employee	26 (6.2)
Marital status mothers	Single	21 (5)
	Married	280 (66.7)
	Divorced	86 (20.5)
	Widowed	33 (7.9)
Education of mother	Primary education	146 (34.8)
	Secondary education and above	274 (65.2)
Ethnicity	Hadiya	190 (45.2)
	Kembata	84 (20)
	Tigre	25 (6)
	Gurage	17 (4)
	Silte	53 (12.6)
	Wolaita	11 (1.5)
Residence	Urban	210 (50)
	Rural	210 (50)

**Table 3:** Obstetric and clinical characteristics of the study participants attending antenatal care at Public Hospitals in Hadiya Zone, Southern Ethiopia: August 2019-December 2020 (*n* = 420).

Variables	Categories	n (%)
Family history of DM	Yes No	167 (39.8) 253 (60.2)
Previous history of GDM	Yes No	100 (23.8) 320 (76.2)
Family history of type II DM	Yes No	167 (39.8) 253 (60.2)
Parity	Nulliparous Para one Multipara Grand multipara (>5)	0 (0) 140 (33.3) 180 (42.8) 100 (23.8)
Gravidity	One Two Three Four Five or more	41 (9.8) 99 (23.6) 41 (9.8) 113 (26.9) 126 (30)
MUAC	MUAC ≥ 28 cm MUAC < 28 cm	188 (44.8) 232 (55.2)
Blood pressure	Systolic blood pressure (mmHg)	Yes 204 (48.6)
		No 216 (51.4)
	Diastolic blood pressure (mmHg)	Yes 204 (48.6)
		No 216 (51.4)
Blood glucose level, 2 h blood glucose (OGTT) (mg/dl	≥140-199 mg/dl	110 (26.2)
	<140 mg/dl	310 (73.8)
Hemoglobin	Normal (Hb ≥ 11 g/dl) Anemia (Hb < 11 g/dl)	309 (73.6) 111 (26.4)
Previous cesarean section	Yes No	94 (22.4) 326 (77.6)
Previous abortion	Yes No	238 (56.6) 182 (43.3)
History of having a macrocosmic baby	Yes No	53 (12.6) 367 (87.4)
Preterm labor	Yes No	167 (39.7) 253 (60.3)
Previous stillbirth	Yes No	120 (28.6) 300 (71.4)
Birth weight of the previous child	Less than 2.5 kg (underweight)	73 (17.4)
	2.5-3.9 kg (normal)	294 (70)
	4 kg or more(overweight)	53 (12.6)
Gestational age in weeks	2^nd^ trimester (24-28 wks.) 3^rd^ trimester (24-28 wks.)	302 (71.9) 118 (28.1)

**Table 4:** Behavioral and life characteristics of the study participants attending antenatal care at public hospitals in Hadiya Zone, Southern Ethiopia: August 2019-December 2020 (*n* = 420).

Variables	Categories	*n* (%)
Level of physical activity	High Moderate Low	10 (2.4) 30 (7.1) 380 (90.5)
Dietary diversity status	Inadequate (<5) Adequate (≥5)	290 (69) 130 (31)
Antenatal depression	Yes No	30 (7.1) 390 (92.9)
Alcohol intake	Yes No	73 (17.4) 347 (82.6)
Coffee intake	Yes No	390 (92.8) 30 (7.2)
Khat chewing	Yes No	26 (6.2) 394 (93.8)

**Table 5:** The final multivariable binary logistic regression model showing risk factors independently associated with gestational diabetes mellitus among the person of working age in Hadiya Zone public Hospitals, Southern Ethiopia: August 2019-December 2020 (*n* = 420).

Variable	Gestational diabetes mellitus	
	Yes GDM	No GDM	COR (95% CI)	AOR (95% CI)	*P* value
Residency					
Urban	75 (35.7)	135 (64.3)	0.360 (0.227, 0.570)	2.181 (1.274, 3.733)^∗^	0.0004
Rural	35 (16.7)	175 (83.3)	1		
Education of mothers					
Primary education	47 (32.2)	99 (67.8)	3.57 (1.017, 2.486)	2.286 (1.396, 3.745)^∗^	0.001
Secondary education and above	63 (23)	211 (77)	1		
Previous history abortion					
Yes	100 (42)	138 (58)	1		
No	10 (5.5)	172 (94.5)	0.080 (0.040, 0.160)	0.097 (0.048, 0.196) ^∗^	0.001
Gestational age in weeks					
2^nd^ trimesters (24-28 wks.)	88 (29.1)	214 (70.9)	1		
3^rd^ trimesters (29-32wks)	22 (18.6)	96 (81.4)	5.950 (0.329, 0.943)	0.393 (0.213, 0.723)^∗^	0.003
Coffee drink					
Yes	99 (25.4)	291 (74.6)	1		
No	11 (36.7)	19 (63.3)	1.702 (0.783, 3.700)	2.704 (1.044, 7.006)^∗^	0.041
Dietary diversity status					
Inadequate (<5)	54 (18.6)	236 (81.4)	1		
Adequate (≥5)	56 (43.1)	74 (56.9)	0.302 (0.192, 0.477)	2.740 (1.585, 4.739)^∗^	0.001

1: reference, ^∗^ shows the variable significance at *P* value ≤ 0.05 in multivariable analysis.

## Data Availability

The datasets used and/or analyzed during the current study are available upon request from the corresponding author.

## Abbreviations

ANC: Antenatal care
AOR: Adjusted odd ratios
BP: Blood pressure
BSc: Bachelor of Science
DBP: Diastolic blood pressure
DM: Diabetes mellitus
EPDS: Edinburgh Postnatal Depression Scale
ETB: Ethiopian Birr
FANTA: Food and Nutrition Technical Assistance
GA: Gestational age
GDM: Gestational diabetes mellitus
IPAQ: International Physical Activity Questioner
IRB: Institutional review board
MDDS: Minimum dietary diversity score
MUAC: Middle upper arm circumference
MUAC: Middle upper arm circumference
NCDs: Noncommunicable diseases
OGTT: Oral glucose tolerance test
PPS: Probability proportional to sample size
SBP: Systolic blood pressure
SNNPR: Southern Nation Nationality People Regional State
SPSS: Statistical Package for Social Science
SS: Systematic sampling
WCU: Wachemo University
WHO: World Health Organization.

## Additional Points

*Copyright.* Before the article can be processed, all authors must sign the “submission form” agreement and we all agreed to submit the manuscript in your journal.
